# COVID-19 and Rates of Cancer Diagnosis in the US

**DOI:** 10.1001/jamanetworkopen.2024.32288

**Published:** 2024-09-06

**Authors:** Todd Burus, Feitong Lei, Bin Huang, W. Jay Christian, Pamela C. Hull, Amanda R. Ellis, Svetla Slavova, Thomas C. Tucker, Krystle A. Lang Kuhs

**Affiliations:** 1 Markey Cancer Center, University of Kentucky, Lexington; 2 Division of Cancer Biostatistics, College of Medicine, University of Kentucky, Lexington; 3 Kentucky Cancer Registry, Markey Cancer Center, University of Kentucky, Lexington; 4 Department of Epidemiology & Environmental Health, College of Public Health, University of Kentucky, Lexington; 5 Department of Behavioral Science, College of Medicine, University of Kentucky, Lexington; 6 Department of Biostatistics, College of Public Health, University of Kentucky, Lexington; 7 Kentucky Injury Prevention & Research Center, University of Kentucky, Lexington

## Abstract

**Importance:**

US cancer diagnoses were substantially lower than expected during the COVID-19 pandemic in 2020. A national study on the extent to which rates recovered in 2021 has not yet been conducted.

**Objective:**

To examine observed vs expected cancer rate trends for January 2020 to December 2021.

**Design, Setting, and Participants:**

This cross-sectional, population-based study of cancer incidence trends used the Surveillance, Epidemiology, and End Results 22 (SEER-22) Registries Database, which covers 47.9% of the US population. Included individuals were those with an invasive cancer diagnosis reported to registries included in SEER-22 between January 1, 2000, and December 31, 2021.

**Exposures:**

Age, sex, race and ethnicity, urbanicity, and stage at diagnosis.

**Main Outcomes and Measures:**

Expected cancer incidence rates were measured for the COVID-19 pandemic years of 2020 and 2021 from prepandemic trends using ensemble forecasting methods. Relative difference between observed and expected cancer incidence rates and numbers of potentially missed cases were measured.

**Results:**

The SEER-22 registries reported 1 578 697 cancer cases in 2020 and 2021, including 798 765 among male individuals (50.6%) and 909 654 among persons aged 65 years or older (57.6%). Observed all-sites cancer incidence rates were lower than expected by 9.4% in 2020 (95% prediction interval [PI], 8.5%-10.5%), lower than expected by 2.7% in 2021 (95% PI, 1.4%-3.9%), and lower than expected by 6.0% across both years combined (95% PI, 5.1%-7.1%), resulting in 149 577 potentially undiagnosed cancer cases (95% PI, 126 059-176 970). Of the 4 screening-detected cancers, only female breast cancer showed significant recovery in 2021, exceeding expected rates by 2.5% (95% PI, 0.1%-4.8%), while significant reductions remained for lung cancer (9.1% lower than expected; 95% PI, 6.4%-13.2%) and cervical cancer (4.5% lower than expected; 95% PI, 0.4%-8.0%), particularly for early stage at diagnosis. Rates of all-sites cancer incidence returned to prepandemic trends in 2021 among female individuals, persons aged younger than 65 years, and persons of non-Hispanic Asian and Pacific Islander race and ethnicity.

**Conclusions and Relevance:**

This population-based cross-sectional study of US cancer incidence trends found that rates of diagnosis improved in 2021 but continued to be lower than expected, adding to the existing deficit of diagnosed cases from 2020. Particular attention should be directed at strategies to immediately increase cancer screenings to make up lost ground.

## Key Points

**Question:**

To what extent did disruptions to the diagnosis of cancer in 2020 resolve during the second year of the COVID-19 pandemic in the US?

**Findings:**

This population-based cross-sectional study found that rates of observed cancer diagnoses improved in 2021 but still remained a significant 2.7% lower than expected. Among screening-detected cancers, female breast cancer showed significant rate recovery in 2021, colorectal cancer returned to prepandemic trends, and significant reductions in diagnoses remained for lung and cervical cancers.

**Meaning:**

This study suggests that cancer cases in the US continued to be underdiagnosed during the second year of the COVID-19 pandemic.

## Teaser

This cross-sectional study examines trends in expected vs observed rates of cancer diagnoses in the US during the first 2 years of the COVID-19 pandemic.

## Introduction

The association of COVID-19 pandemic–related disruptions with the timely diagnosis of US cancer cases in 2020 has been well documented.^1,2,3,4,5^ In a previous study, we estimated that rates of all-sites cancer incidence were 13.0% lower than expected during the first 10 months of the pandemic (March-December 2020), with even greater differences seen among certain cancer sites, stages at diagnosis, and population subgroups.^1^ If, and to what extent, cancer rates recovered in 2021 is still unknown. In this study, we used observed cancer incidence rates from an authoritative collection of US cancer registries to assess the state of cancer diagnoses during the second year of the COVID-19 pandemic and better understand the lasting association of COVID-19 with cancer burden in the US.

## Methods

### Data Sources

We obtained information on cancer incidence from the National Cancer Institute’s Surveillance, Epidemiology, and End Results 22 (SEER-22) Registries Database, November 2023 Submission.^6^ The SEER-22 database includes cancer cases from 22 population-based central cancer registries and covers approximately 47.9% of the US population. Annual invasive cancer incidence rates per 100 000 persons were calculated for January 2000 through December 2021 and age-adjusted to the 2000 US standard population using SEER*Stat statistical software, version 8.4.3.^7^ Additional incidence rates were obtained by month of diagnosis for January 2017 through December 2021. Information about patient sex, age, race and ethnicity, urbanicity, and cancer stage at diagnosis was also extracted. Incidence rates from 2000 to 2019 were used to estimate expected incidence rates in 2020 and 2021. Characteristics of cases diagnosed in 2020 and 2021 are detailed in the Results. This study was deemed exempt from review by the University of Kentucky institutional review board because it was not human participants research; the need for informed consent was waived because data were deidentified. We followed the Strengthening the Reporting of Observational Studies in Epidemiology (STROBE) reporting guideline.

### Study Design

In this population-based cross-sectional study, we identified patients with any invasive cancer diagnosis between 2000 and 2021 to calculate annual all-sites cancer incidence rates. Monthly rates were also calculated for all months between January 2017 and December 2021. Only cases with available month of diagnosis were included in calculations (missing month of diagnosis occurred in <1% of cases diagnosed between 2000 and 2021). We calculated incidence rates for additional sites and site groupings consistent with the *International Classification of Diseases for Oncology, Third Edition* Site Recode/World Health Organization 2008 definition.^8^ Rates were obtained for sites included in the US Cancer Statistics’ top 10 cancers by rates of new cases for 2020: breast (females only); prostate; lung and bronchus; colon and rectum; corpus and uterus, not otherwise specified; melanoma of the skin; urinary bladder; non-Hodgkin lymphoma; kidney and renal pelvis; and pancreas.^9^ We also calculated rates for cervix uteri to include all 4 cancers with a US Preventive Services Task Force (USPSTF) high-grade recommendation for screening (alongside female breast cancer, lung cancer, and colorectal cancer). Incidence rates were calculated among the total population as well as by patient sex (female or male), age (<65 years or ≥65 years), race and ethnicity (Hispanic, non-Hispanic Asian and Pacific Islander, non-Hispanic Black, or non-Hispanic White), urbanicity (metropolitan or nonmetropolitan), and cancer stage at diagnosis. Race and ethnicity data were collected to assess whether disparities existed in missed cancer diagnoses between different racial or ethnic groups; data were classified according to the SEER program’s “Race and origin (recommended by SEER)” variable, which algorithmically recodes detailed race and Hispanic origin for patients based on available records and using imputation of Hispanic origin as needed.^10^ We did not calculate rates for non-Hispanic American Indian or Alaska Native or non-Hispanic unknown race individuals due to low case counts. Urbanicity was determined by Rural Urban Continuum Code (RUCC) 2013 for county of residence, with metropolitan defined as RUCCs 1 to 3 and nonmetropolitan defined as RUCCs 4 to 9.^11^ We used the Combined Summary Stage (2004+) variable to classify stage at diagnosis.^6^ Early stage was defined as localized stage at diagnosis, and late stage was defined as regional or distant stage at diagnosis. Cases from January 2000 through December 2003 were excluded from stage at diagnosis incidence rate calculations due to unavailability of stage classification. Staging was collected only for all-sites cancer, female breast cancer, lung cancer, colorectal cancer, and cervical cancer cases.

### Statistical Analysis

We estimated expected annual cancer incidence rates for 2020 and 2021 as a linear combination of 2 different modeling approaches. First, we estimated piecewise log-linear trends in annual age-adjusted cancer incidence rates from 2000 to 2019 using Joinpoint software, version 5.1.0.^12^ Trends were fit using weighted bayesian information criteria selection and parametric confidence intervals, with a maximum of 3 segments that included at least 4 data points per segment. We extracted parameter estimates for the linear model on the last segment to construct a weighted linear regression model for projections. We simulated 10 000 projections of age-adjusted incidence rates in 2020 and 2021 to calculate bootstrap expected rates with pointwise 95% prediction intervals (PIs). Additional methodologic details on the first model can be found in the eMethods in Supplement 1.

Next, we used monthly age-adjusted incidence rates from January 2017 through December 2021 to fit autoregressive integrated moving average (ARIMA) models.^13^ Deviations from previous trends were incorporated into the ARIMA models using exogenous regressors.^13,14^ Postulated deviations included a sudden decrease in rates followed by a rapid return to previous trends (pulse impact) for March through May 2020 and a sustained decrease in rates (step change impact) from June 2020 through December 2021. Once an appropriate ARIMA model was fit to a rate trend, observed monthly incidence rates from January 2017 through December 2019 were used to project monthly expected incidence rates for January 2020 through December 2021. Projected monthly rates were aggregated by year to estimate expected annual age-adjusted incidence rates. Additional details on the second model can be found in the eMethods in Supplement 1 and the previous study by Burus et al.^1^

We combined estimates from these models into an ensemble model using a process known as linear pooling with equal weights.^15,16^ Under this method, ensemble point estimates were calculated as the mean of individual point estimates. To construct 95% PIs for ensemble point estimates, we pooled the 10 000 simulated projections from both component models and calculated the 2.5th and 97.5th percentiles.

Expected and observed incidence rates for the combined period of 2020 and 2021 were calculated as the mean of annual expected or observed incidence rates. We calculated relative differences between observed and expected incidence rates for each period (2020, 2021, and 2020-2021 combined) using the formula (observed rate − expected rate)/expected rate. To provide additional interpretive context for some of our findings, we calculated rough estimates for the number of potentially undiagnosed cancer cases nationally by multiplying the absolute difference between observed and expected incidence rates by the 2000 US standard population denominator of 274 633 642 (or 137 316 821 for sex-specific cancer sites).

Statistical significance from formal tests was assessed at *P* < .05, and all hypotheses were 2-sided. Observed rates were deemed significantly different from expected rates when the 95% PI of the relative difference did not contain 0. All analyses were performed in the R statistical programming language, version 4.3.2 (R Project for Statistical Computing).

## Results

### Population Cancer Rates

This study included a total of 1 578 697 cancer cases reported by SEER-22 cancer registries between January 2020 and December 2021 (Table 1).^6^ Of these, 756 221 (47.9%) were diagnosed in 2020, with an age-adjusted incidence rate of 408.0 cases per 100 000 population. The remaining 822 476 cases (52.1%) were diagnosed in 2021, with an age-adjusted incidence rate of 438.3 cases per 100 000 population. Observed cases included 798 765 among male individuals (50.6%) and 779 932 among female individuals (49.4%), 909 654 among persons aged 65 years or older (57.6%), 214 827 among Hispanic individuals (13.6%), 97 658 among non-Hispanic Asian and Pacific Islander individuals (6.2%), 171 427 among non-Hispanic Black individuals (10.9%), 1 061 592 among non-Hispanic White individuals (67.2%), and 1 394 152 among persons living in metropolitan counties (88.3%). Early and late stage at diagnosis occurred at almost identical frequencies (717 100 and 716 515, respectively).

**Table 1.** Study Population, All-Sites Cancer Cases, January 2019 to December 2021^a^

Characteristic	No. (%)		
	2019 (n = 830 007)	2020 (n = 759 810)	2021 (n = 825 645)
Included^b^	825 327 (99.4)	756 221 (99.5)	822 476 (99.6)
Missing month	4680 (0.6)	3589 (0.5)	3169 (0.4)
Sex			
Male	418 226 (50.7)	383 971 (50.8)	414 794 (50.4)
Female	407 101 (49.3)	372 250 (49.2)	407 682 (49.6)
Age, y			
<65	356 679 (43.2)	322 319 (42.6)	346 724 (42.2)
≥65	468 648 (56.8)	433 902 (57.4)	475 752 (57.8)
Race and ethnicity			
Hispanic (all races)	109 433 (13.3)	101 418 (13.4)	113 409 (13.8)
Non-Hispanic Asian and Pacific Islander	49 513 (6.0)	45 035 (6.0)	52 623 (6.4)
Non-Hispanic Black	89 534 (10.8)	81 176 (10.7)	90 251 (11.0)
Non-Hispanic White	563 286 (68.3)	514 150 (68.0)	547 442 (66.6)
Other non-Hispanic race^c^	13 561 (1.6)	14 442 (1.9)	18 751 (2.3)
Urbanicity			
Metropolitan	727 700 (88.2)	666 581 (88.1)	727 571 (88.5)
Nonmetropolitan	97 010 (11.8)	89 103 (11.8)	94 300 (11.5)
Unknown	617 (0.1)	537 (0.1)	605 (0.1)
Stage			
Early	377 410 (45.7)	335 624 (44.4)	381 476 (46.4)
Late	368 224 (44.6)	347 262 (45.9)	369 253 (44.9)
In situ or unknown	79 693 (9.7)	73 335 (9.7)	71 747 (8.7)

^a^ Data from the National Cancer Institute’s Surveillance, Epidemiology, and End Results 22 (SEER-22) Registries Database, November 2023 submission for 2000-2021.^6^

^b^ Denominator used for calculating subgroup percentages.

^c^ Other non-Hispanic race includes American Indian or Alaska Native and unknown.

**Figure 1.**
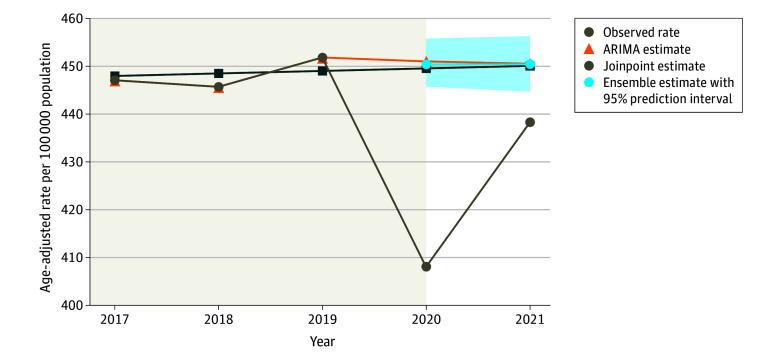
All-Sites Cancer Incidence Rates in the US for January 2017 to December 2021 Time series plot of annual observed all-sites cancer incidence rates. Expected rate estimates in the absence of the COVID-19 pandemic are displayed, showing comparison of autoregressive integrated moving average (ARIMA) estimates, Joinpoint estimates, and linear pooling with equal weights ensemble estimates. The shaded area indicates the 95% prediction interval of the expected rates from the ensemble model.

Using the final ensemble model, we estimated that all-sites cancer incidence was a significant 9.4% lower than expected in 2020 (95% PI, 8.5%-10.5%) and 2.7% lower in 2021 (95% PI, 1.4%-3.9%), resulting in a significant rate reduction of 6.0% across both years (95% PI, 5.1%-7.1%) (Table 2 and Figure 1). This equates to a total of 149 577 potentially undiagnosed cancer cases during the first 2 years of the COVID-19 pandemic (95% PI, 126 059-176 970), with 33 226 potentially undiagnosed cases occurring in 2021 (95% PI, 17 513-49 463).

**Table 2.** Potentially Missed All-Sites Cancer Cases by Stage at Diagnosis and Period, 2020-2021^a^

Stage at diagnosis and period	Observed rate^b^	Expected rate (95% PI)	Relative difference (95% PI), %	Potential missed cases (95% PI)
All stages				
2020	408.0	450.4 (445.7 to 455.8)	−9.4 (−10.5 to −8.5)	116 350 (103 551 to 31 117)
2021	438.3	450.4 (444.7 to 456.3)	−2.7 (−3.9 to −1.4)	33 226 (17 513 to 49 463)
Combined	423.2	450.4 (446.1 to 455.4)	−6.0 (−7.1 to −5.1)	149 577 (126 059 to 176 970)
Early stage^c^				
2020	180.4	204.8 (201.1 to 208.6)	−11.9 (−13.5 to −10.3)	67 109 (56 808 to 77 227)
2021	202.7	205.3 (200.4 to 209.6)	−1.3 (−3.3 to 1.2)	7132 (−6385 to 18 878)
Combined	191.6	205.1 (201.5 to 208.2)	−6.6 (−8.0 to −4.9)	74 151 (54 390 to 91 470)
Late stage^d^				
2020	188.0	200.9 (196.8 to 204.8)	−6.4 (−8.2 to −4.5)	35 375 (24 158 to 46 031)
2021	197.5	200.3 (195.9 to 205.1)	−1.4 (−3.7 to 0.8)	7770 (−4300 to 20 895)
Combined	192.8	200.6 (197.2 to 204.6)	−3.9 (−5.8 to −2.3)	43 145 (24 534 to 64 767)

Abbreviation: PI, prediction interval.

^a^ Data from the National Cancer Institute’s Surveillance, Epidemiology, and End Results 22 (SEER-22) Registries Database, November 2023 submission for 2000-2021.^6^

^b^ Rates given per 100 000 people in the population and age-adjusted to the 2000 US standard population.

^c^ Early stage at diagnosis defined as localized stage only.

^d^ Late stage at diagnosis defined as regional or distant stage.

**Figure 2.**
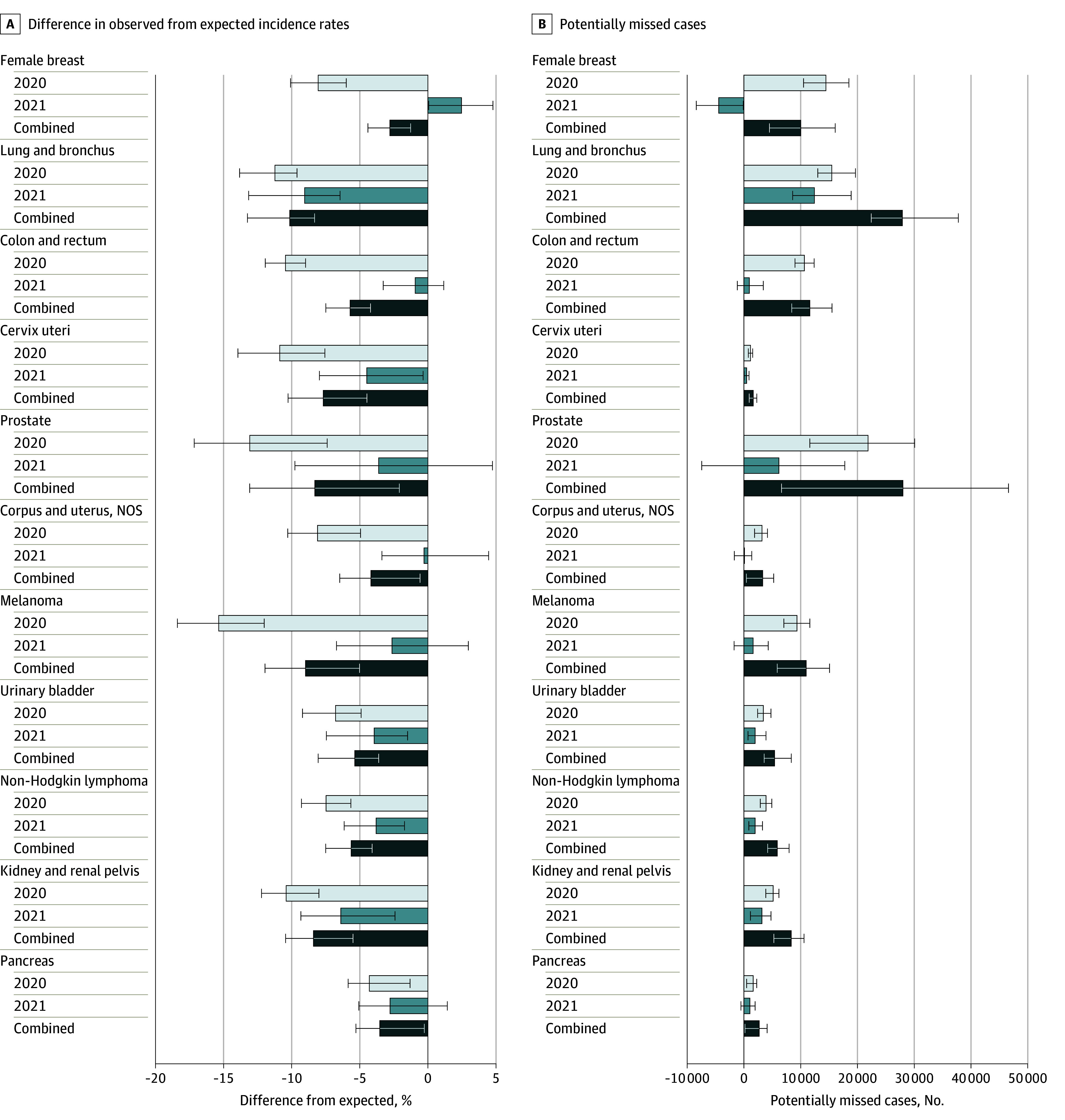
Percentage Difference in Observed From Expected Incidence Rates and Potentially Missed Cases, by Site and Period, January 2020 to December 2021 Error bars indicate the 95% prediction intervals. NOS indicates not otherwise specified.

We also estimated observed incidence rates to be significantly lower than expected in 2020 for all 11 of the specific cancer sites and groupings examined (Figure 2; eTable 1 in Supplement 1). Significant reductions remained for 5 of the 11 sites in 2021 (lung and bronchus; cervix uteri; urinary bladder; non-Hodgkin lymphoma; and kidney and renal pelvis). Continued lower-than-expected incidence rates of lung cancer diagnoses were notable (9.1%; 95% PI, 6.4%-13.2%), as were rates of cervical cancer diagnoses (4.5%; 95% PI, 0.4%-8.0%). Rates of female breast cancer were estimated to significantly exceed expected rates in 2021 (2.5% higher; 95% PI, 0.1%-4.8%). Nevertheless, we estimated that observed incidence rates across all examined sites fell short of combined expected rates during the first 2 years of the COVID-19 pandemic.

### Population Cancer Rates by Stage at Diagnosis

All-sites cancer incidence rates by stage at diagnosis were estimated to be significantly lower than expected for both early- and late-stage disease in 2020, with significantly lower rates among early-stage diagnoses (11.9%; 95% PI, 10.3%-13.5%) than among late-stage diagnoses (6.4%; 95% PI, 4.5%-8.2%) (Table 2). In 2021, we estimated no significant disruption to either early-stage all-sites diagnoses (1.3% lower; 95% PI, 3.3% lower to 1.2% higher) or late-stage all-sites diagnoses (1.4% lower; 95% PI, 3.7% lower to 0.8% higher).

**Figure 3.**
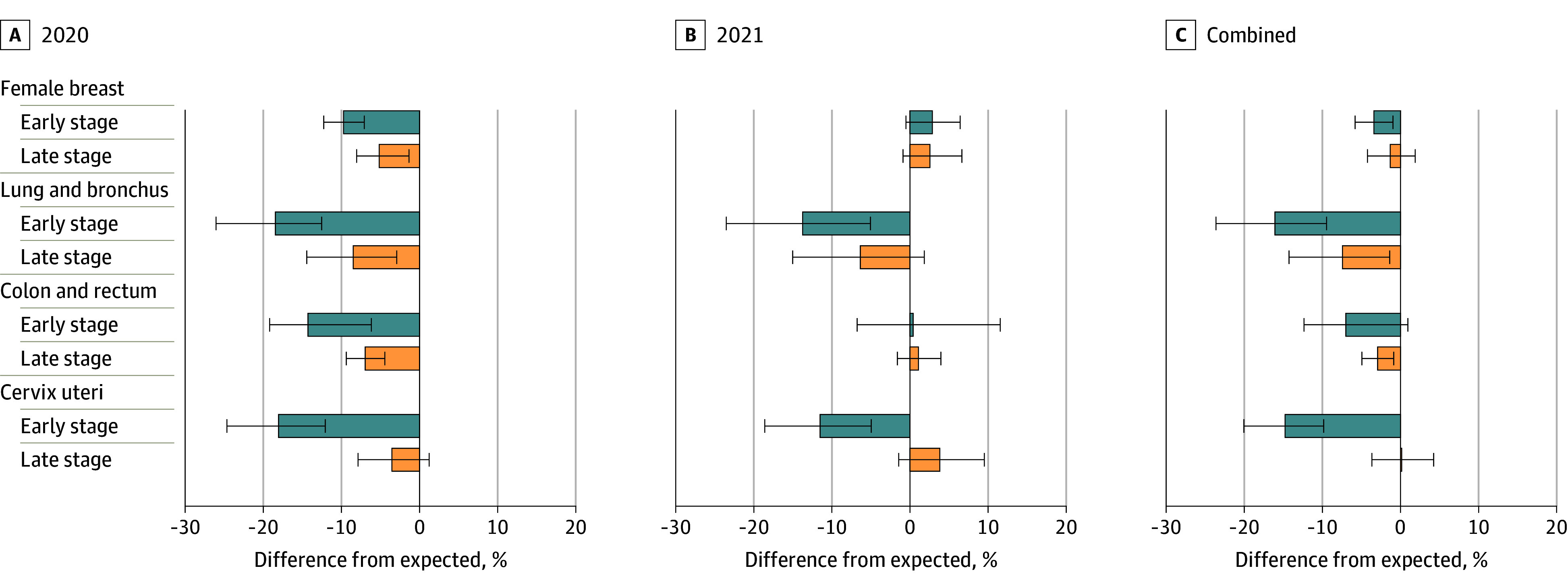
Percentage Difference in Observed From Expected Incidence Rates for Screening-Detected Cancers, by Site, Stage, and Period, January 2020 to December 2021 Error bars indicate the 95% prediction intervals.

For all cancers with USPSTF high-grade screening recommendations (female breast, lung, colorectal, and cervical), both early- and late-stage diagnoses were significantly lower than expected in 2020 with the exception of late-stage cervical cancer (Figure 3; eTable 2 in Supplement 1). In 2021, significant rate reductions persisted for only early-stage diagnoses of lung cancer (13.7%; 95% PI, 5.1%-23.5%) and cervical cancer (11.5%; 95% PI, 4.9%-18.6%). There was some suggestion that late-stage diagnoses of breast, colorectal, and cervical cancers exceeded expected rates in 2021; however, none of these reached statistical significance.

### Population Subgroup Cancer Rates

Every subgroup categorized by age, race and ethnicity, sex, or urbanicity experienced significant disruptions to all-sites cancer incidence in 2020 (eFigure 1 in Supplement 1). By 2021, all-sites cancer incidence returned to prepandemic levels among female individuals, persons younger than 65 years, and non-Hispanic Asian and Pacific Islander individuals. However, all-sites cancer incidence remained significantly lower than expected among male individuals (3.5%; 95% PI, 2.2%-5.0%), persons aged 65 years or older (3.3%; 95% PI, 2.0%-4.6%), and Hispanic individuals (2.9%; 95% PI, 1.3%-4.4%), non-Hispanic Black individuals (2.2%; 95% PI, 0.7%-3.7%), and non-Hispanic White individuals (3.6%; 95% PI, 2.2%-4.8%). No significant between-group differences were found for overall or site-specific cancer diagnoses in 2020 or 2021 (eFigures 1-5 in Supplement 1). Considering the interaction between sex and age, we found that female individuals younger than 65 years experienced significantly less disruption in rates of all-sites cancer incidence in 2021 and 2020-2021 combined than male individuals of either age group (eFigure 6 in Supplement 1).

## Discussion

Several prior studies reported significantly lower-than-expected rates of cancer diagnoses in the US in 2020 due to disruptions caused by the COVID-19 pandemic.^1,2,3,4,5^ In this study, we offer the first analysis, to our knowledge, from high-quality US cancer registry data on whether—and to what extent—cancer incidence rates recovered to pre–COVID-19 pandemic trends in 2021. Despite experiencing less disruption than in 2020, we estimate that overall diagnoses of new cancer cases still fell short of expected levels during the second year of the pandemic. Among specific cancer sites, only female breast cancer showed significant evidence of making up for previously missed cases, while significant deficits remained for diagnoses of lung, cervical, bladder, and kidney cancers and non-Hodgkin lymphoma. Continued reductions in early-stage lung and cervical cancer rates are particularly concerning, as is the nonsignificant suggestion of increased rates of late-stage diagnoses among 3 of the 4 screening-detected cancers. Overall, our findings suggest that pandemic-related disruptions to cancer diagnoses in the US lasted well beyond the first few months of 2020.

Had all pandemic-related disruptions to cancer diagnoses from 2020 resolved then, ideally, all undiagnosed 2020 cases—as well as all new 2021 cases—would have been diagnosed in 2021, resulting in greater-than-expected 2021 incidence rates. The null hypothesis of our analysis tested only whether rates returned to prepandemic trends. Unfortunately, even this weaker assumption failed to hold for overall cancer diagnoses and for many individual cancer sites. Of greatest concern are the continued disruptions occurring among early-stage lung and cervical cancer diagnoses. Unlike colorectal cancer—for which we estimated rate recovery in 2021 after significant reductions in 2020—early-stage lung cancer and cervical cancer incidence rates remained significantly below expected in each of the first 2 years of the pandemic. These differences may suggest prolonged reductions in cancer screening among these sites.^17,18^ Zhang et al^19^ documented substantial cervical cancer screening delays in 2020, as did Alba et al for 2021.^20^ Doan et al^21^ reported similar reductions in lung cancer screening appointments between March 2020 and April 2022. Extended wait times for women’s health appointments and decisions to defer new enrollment in lung cancer screening may have compounded these issues.^22,23^ Missed or delayed cancer screenings may also lead to an increase in late-stage diagnoses (upstaging) and poorer survival. No definitive evidence of upstaging can be found in our analysis, although we did estimate nominally higher-than-expected rates of late-stage diagnoses for 3 of the 4 screening-detected cancers in 2021. It is crucial that immediate and intentional efforts be made to increase cancer screenings among eligible populations.

Several studies of delayed medical care during the first year of the COVID-19 pandemic highlighted fears associated with COVID-19 infection as a significant contributing factor, particularly among female and younger individuals.^24,25,26,27^ Our findings of cancer incidence rate recovery in 2021 among females and individuals younger than 65 years suggest a lessening of these fears. Analyzing monthly all-sites cancer incidence data, we found substantial increases in rates of diagnosis in March and April 2021, with younger females experiencing their 2 highest monthly incidence rates between January 2017 and December 2021 during this period (eFigure 7 in Supplement 1). March and April 2021 correspond to when the first COVID-19 vaccines became widely available among the general adult population in the US.^28^ Further research is called for into the effect of COVID-19 vaccine availability and return to medical care among US adults.

Although a strength of this study is its use of high-quality, population-based cancer registry data, the timing and findings of this analysis raise additional concerns about the US public health infrastructure. As the COVID-19 pandemic winds down, it is necessary to learn lessons that will improve responses to future public health crises. One lesson that can be learned from our work is the need for improvements in national cancer surveillance. The ongoing disruptions to lung and cervical cancer diagnoses we have found are only coming to light 2 years after they occurred. That disruptions in lung and cervical cancer diagnoses may be the result of prolonged decreases in screening is something that could not be observed sooner because of a lack of regular and consistent nationwide screening surveillance. Investments must be made to increase the speed, quality, and consistency of cancer-related data collection and reporting, as delayed awareness of systemic problems can result in additional and unnecessary losses among the population.

### Limitations

Certain limitations exist within this study. First, projecting expected cancer incidence rates across 2 years is subject to various uncertainties. Our use of 2 projection models and a well-established method for integrating both results helps to better account for these uncertainties in our estimates. Moreover, the conservative 95% PIs that arise from this method provide additional assurance that significant findings are truly worth attention. Limitations within the ensemble model and its underlying components have been addressed previously by Wang et al,^16^ Chen et al,^29^ and Burus et al.^1^ Second, actual cancer incidence rates may be higher than stated in 2020 and 2021 due to delays in case reporting. Nevertheless, wide 95% PI error bounds within the ensemble model mean that our primary conclusions are unlikely to be meaningfully affected if data are revised. In addition, our estimates of missed diagnoses in 2020 provide meaningful updates to previous studies through the inclusion of cases from 2020 that were not yet reported by the release of earlier datasets. Third, cases without an available month of diagnosis were excluded from analysis, which accounts for less than 1% of cases during the years considered, with no evidence of particular months having greater amounts of incomplete data. Fourth, estimates of potentially undiagnosed cancer cases do not account for changing population age distributions over time. The use of conservative 95% PIs, however, provides a sufficient range of uncertainty to cover possible discrepancies. A sensitivity analysis using age group stratification can be found in the eMethods in Supplement 1. Fifth, expected incidence rates for 2021 were not updated to include individuals whose cancer went undiagnosed in 2020. As a result, true rate reductions in 2021 are potentially greater than estimated in this study. Sixth, no attempt at causal inference was made in this study, and as such, it is possible that observed rate reductions could be due to natural changes in cancer diagnoses that coincided with the COVID-19 pandemic. Our use of trends from multiple prepandemic years helps lessen, but not eliminate, this possibility.

## Conclusions

In this population-based cross-sectional study of US cancer incidence trends in 2020 and 2021, rates of US cancer diagnoses continued to be lower than expected during the second year of the COVID-19 pandemic, which added to the existing deficit of diagnosed cases from 2020. Variations in recovery occurred with respect to site, stage at diagnosis, and patient demographics, highlighting specific areas to target for clinical and community interventions. Particular attention should be directed at strategies to immediately increase cancer screenings to make up lost ground and prevent a future surplus of late-stage diagnoses.

## Notes

Supplement 1. **eMethods.** Supplemental Description of Methods **eTable 1.** Potentially Missed Cancer Cases, by Site and Period, 2020-2021, SEER 22 Database, 2001-2021 **eTable 2.** Potentially Missed Screening-Detected Cancer Cases by Stage at Diagnosis and Period, 2020-2021, SEER 22 Database, 2001-2021 **eFigure 1.** Percentage Difference in Observed From Expected All Sites Cancer Incidence Rates, by Population Subgroup and Period, 2020-2021 **eFigure 2.** Percentage Difference in Observed From Expected Incidence Rates, by Urbanicity, Site, and Period, 2020-2021 **eFigure 3.** Percentage Difference in Observed From Expected Incidence Rates, by Age, Site, and Period, 2020-2021 **eFigure 4.** Percentage Difference in Observed From Expected Incidence Rates, by Race and Ethnicity, Site, and Period, 2020-2021 **eFigure 5.** Percentage Difference in Observed From Expected Incidence Rates, by Sex, Site, and Period, 2020-2021 **eFigure 6.** Percentage Difference in Observed From Expected Incidence Rates, by Sex, Age, Site, and Period, 2020-2021 **eFigure 7.** Monthly Age-Adjusted All Cancer Sites Incidence Rates, by Sex and Age, January 2017-December 2021
eReferences.

Supplement 2.
Data Sharing Statement
