# Supplementary material for: COVID-19 and Rates of Cancer Diagnosis in the US
Source: JAMA Netw Open. 2024 Sep 6;7(9):e2432288. doi: 10.1001/jamanetworkopen.2024.32288 (PMC11380103; doi:10.1001/jamanetworkopen.2024.32288)
Supplement: Supplement 1. — eMethods. Supplemental Description of Methods eTable 1. Potentially Missed Cancer Cases, by Site and Period, 2020-2021, SEER 22 Database, 2001-2021 eTable 2. Potentially Missed Screening-Detected Cancer Cases by Stage at Diagnosis and Period, 2020-2021, SEER 22 Database, 2001-2021 eFigure 1. Percentage Difference in Observed From Expected All Sites Cancer Incidence Rates, by Population Subgroup and Period, 2020-2021 eFigure 2. Percentage Difference in Observed From Expected Incidence Rates, by Urbanicity, Site, and Period, 2020-2021 eFigure 3. Percentage Difference in Observed From Expected Incidence Rates, by Age, Site, and Period, 2020-2021 eFigure 4. Percentage Difference in Observed From Expected Incidence Rates, by Race and Ethnicity, Site, and Period, 2020-2021 eFigure 5. Percentage Difference in Observed From Expected Incidence Rates, by Sex, Site, and Period, 2020-2021 eFigure 6. Percentage Difference in Observed From Expected Incidence Rates, by Sex, Age, Site, and Period, 2020-2021 eFigure 7. Monthly Age-Adjusted All Cancer Sites Incidence Rates, by Sex and Age, January 2017-December 2021 eReferences. [file jamanetwopen-e2432288-s001.pdf]

Supplementary Online Content

Burus T, Lei F, Huang B, et al. COVID-19 and rates of cancer diagnosis in the US. *JAMA Netw Open.* 2024;7(9):e2432288. doi:10.1001/jamanetworkopen.2024.32288

eMethods. Supplemental Description of Methods

eTable 1. Potentially Missed Cancer Cases, by Site and Period, 2020-2021, SEER 22 Database, 2001-2021

eTable 2. Potentially Missed Screening-Detected Cancer Cases by Stage at Diagnosis and Period, 2020-2021, SEER 22 Database, 2001-2021

eFigure 1. Percentage Difference in Observed From Expected All Sites Cancer Incidence Rates, by Population Subgroup and Period, 2020-2021

eFigure 2. Percentage Difference in Observed From Expected Incidence Rates, by Urbanicity, Site, and Period, 2020-2021

eFigure 3. Percentage Difference in Observed From Expected Incidence Rates, by Age, Site, and Period, 2020-2021

eFigure 4. Percentage Difference in Observed From Expected Incidence Rates, by Race and Ethnicity, Site, and Period, 2020-2021

eFigure 5. Percentage Difference in Observed From Expected Incidence Rates, by Sex, Site, and Period, 2020-2021

eFigure 6. Percentage Difference in Observed From Expected Incidence Rates, by Sex, Age, Site, and Period, 2020-2021

eFigure 7. Monthly Age-Adjusted All Cancer Sites Incidence Rates, by Sex and Age, January 2017-December 2021

eReferences.

This supplementary material has been provided by the authors to give readers additional information about their work.

© 2024 Burus T et al. *JAMA Network Open.*

eMethods. Supplemental Description of Methods

A. Joinpoint regression estimates.

Joinpoint regession estimates of expected incidence rates for 2020 and 2021 were produced based on Joinpoint regression analysis of annual incidence rate trends from 2000 to 2019.[1] In particular, for every cancer site of interest (with sex, race and ethnicity, age, urbanicity, or stage at diagnosis stratification, as applicable) we first fit a piecewise log-linear regression model to cancer incidence rate trends from 2000 to 2019 using the National Cancer Institute's Joinpoint software version 5.1.0. Prospective models were constrained to at most two breaks (referred to as joinpoints) resulting in no more than three distinct linear segments, each of which was required to contain at least four years of data. Individual linear segments were fit using weighted least squares regression with inverse variance weights. The final piecewise regression model was selected based upon the least weighted Bayesian Information Criteria value and parametric 95% confidence intervals were determined.[2]

Once a best Joinpoint regression model was determined, slope and intercept parameters for the linear model defining the final segment were extracted. This segment was guaranteed to cover observed incidence rates for the years of 2016-2019 at a minimum, and give the best estimate of trends in the years leading up to the start of the COVID-19 pandemic. We used these parameters to reconstruct the weighted linear regression model for the final segment in the R statistical programming language (version 4.3.2; R Foundation) and simulated 10,000 projections for expected incidence rates in 2020 and 2021. We estimated a final expected incidence rate for both years using the mean of the simulated projections and determined a 95% prediction interval (PI) by taking the 2.5th and 97.5th percentiles of simulated projections.

B. ARIMA estimates

The autoregressive and integrated moving average (ARIMA) estimates of expected incidence rates for 2020 and 2021 were developed according to the method described previously in Burus et al.[3] For this paper, we sought to estimate monthly age-adjusted incidence rates for January 2020-December 2021 in order to align with the annual age-adjusted estimates from the Joinpoint regression method. To accommodate for adding an additional 12 points to the projection, an additional 12 months of data

© 2024 Burus T et al. *JAMA Network Open.*

(January-December 2017) were added to the pre-pandemic trends. We retained the same postulated pulse impact exogenous regressor as used previously for March-May 2020, and extended the postulated step change impact to include the entire 2021 calendar year. Extending the postulated step change impact allows for testing whether monthly incidence rates for June 2020-December 2021 returned to pre-pandemic trends. Additional exogenous regressors were considered to account for successive waves of COVID-19 infection during 2021, but were not incorporated due to a lack of well-defined periods and a desire to avoid overfitting models.

C. Ensemble estimates (linear pooling with equal weights)

We combined Joinpoint regression and ARIMA estimates to construct a final ensemble estimate of expected incidence rates using the method of linear pooling with equal weights.[4,5] Ensemble point estimates for 2020 and 2021 were calculated as the mean of the combined set of 10,000 simulated Joinpoint projections and 10,000 simulated ARIMA projections. Ensemble 95% PI bounds were calculated as the 2.5th and 97.5th percentiles of the combined Joinpoint and ARIMA simulations.

D. Potentially undiagnosed cases sensitivity analysis

For additional interpretive context, estimates for potentially undiagnosed cancer cases were generated for some of our findings by multiplying the absolute differences between observed and expected incidence rates by the 2000 US Standard Population denominator of 274,633,642 (or 137,316,821 for sex-specific cancer sites). Though this calculation does not account for changes in the underlying age distribution of populations over time, the use of conservative 95% prediction intervals for estimates makes differences negligible. To demonstrate this, we performed a sensitivity analysis comparing our original estimates with estimates generated using our age-stratified findings for the age groups of under age 65 years and age 65 years or older, weighted according to the appropriate proportions of the 2000 US Standard Population (0.874 and 0.126, respectively). The results are presented in the following table. As expected, all adjusted estimates fall within the 95% prediction intervals of the original estimates:

© 2024 Burus T et al. *JAMA Network Open.*

Sensitivity analysis for estimates of potentially undiagnosed cancer cases by site and period

Site	Period	Original Estimate (95% PI)	Adjusted Estimate
All Sites	2020	116350 (103551-131117)	112974
	2021	33226 (17513-49463)	28817
	Combined	149577 (126059-176970)	141790
Female Breast	2020	14441 (10512-18484)	15613
	2021	-4432 (-8380 to -114)	-2961
	Combined	10009 (4495-16079)	12652
Lung and Bronchus	2020	15489 (13016-19643)	15255
	2021	12415 (8595-18893)	12086
	Combined	27904 (22428-37764)	27340
Colon and Rectum	2020	10654 (8991-12364)	10410
	2021	953 (-1157 to 3412)	900
	Combined	11607 (8432-15517)	11310
Cervix Uteri	2020	1173 (786-1557)	1143
	2021	485 (37-893)	432
	Combined	1658 (934-2279)	1574
Prostate	2020	21861 (11602-30095)	22794
	2021	6161 (-7433 to 17764)	6966
	Combined	28022 (6629-46609)	29760
Corpus and Uterus, NOS	2020	3196 (1887-4158)	3160
	2021	113 (-1689 to 1382)	37
	Combined	3308 (439-5247)	3196
Melanoma	2020	9349 (7037-11613)	9242
	2021	1613 (-1718 to 4281)	1623
	Combined	10962 (5875-15088)	10864
Urinary Bladder	2020	3423 (2423-4769)	3351
	2021	1984 (730-3885)	1781
	Combined	5407 (3574-8349)	5132
Non-Hodgkin Lymphoma	2020	3892 (2889-4926)	3569
	2021	1975 (876-3271)	1488
	Combined	5867 (4190-7966)	5056
Kidney and Renal Pelvis	2020	5151 (3857-6170)	4738
	2021	3173 (1147-4773)	2836
	Combined	8324 (5280-10584)	7574
Pancreas	2020	1628 (481, 2257)	1554
	2021	1058 (-520 to 1978)	975
	Combined	2686 (196-4084)	2530

Abbreviations: PI = Prediction Interval; NOS = Not Otherwise Specified

© 2024 Burus T et al. *JAMA Network Open.*

eTable 1. Potentially Missed Cancer Cases, by Site and Period, 2020-2021, SEER 22 Database, 2001-2021[6]

	Period	Observed Rate[a]	Expected Rate (95% PI)	Relative Difference (95% PI)	Potential Missed Cases (95% PI)
Female Breast	2020	120.1	130.6 (127.7-133.5)	-8.1% (-10.1% to -6.0%)	14441 (10512-18484)
	2021	134.1	130.9 (128.0-134.0)	2.5% (0.1%-4.8%)	-4432 (-8380 to -114)
	Combined	127.1	130.7 (128.7-132.9)	-2.8% (-4.4% to -1.3%)	10009 (4495-16079)
Lung and Bronchus	2020	44.6	50.2 (49.3-51.7)	-11.2% (-13.8% to -9.6%)	15489 (13016-19643)
	2021	45.4	49.9 (48.6-52.3)	-9.1% (-13.2% to -6.4%)	12415 (8595-18893)
	Combined	45.0	50.1 (49.1-51.9)	-10.1% (-13.3% to -8.3%)	27904 (22428-37764)
Colon and Rectum	2020	33.2	37.1 (36.5-37.7)	-10.5% (-11.9% to -9.0%)	10654 (8991-12364)
	2021	36.6	36.9 (36.2-37.8)	-0.9% (-3.3% to 1.2%)	953 (-1157 to 3412)
	Combined	34.9	37.0 (36.4-37.7)	-5.7% (-7.5% to -4.2%)	11607 (8432-15517)
Cervix Uteri	2020	7.0	7.9 (7.6-8.1)	-10.9% (-13.9% to -7.6%)	1173 (786-1557)
	2021	7.5	7.9 (7.5-8.2)	-4.5% (-8.0% to -0.4%)	485 (37-893)
	Combined	7.3	7.9 (7.6-8.1)	-7.7% (-10.3% to -4.5%)	1658 (934-2279)
Prostate	2020	105.9	121.8 (114.3-127.8)	-13.1% (-17.2% to -7.4%)	21861 (11602-30095)
	2021	119.6	124.1 (114.2-132.6)	-3.6% (-9.8% to 4.7%)	6161 (-7433 to 17764)
	Combined	112.7	122.9 (115.2-129.7)	-8.3% (-13.1% to -2.1%)	28022 (6629-46609)
Corpus and Uterus, NOS	2020	26.4	28.7 (27.8-29.4)	-8.1% (-10.3% to -4.9%)	3196 (1887-4158)
	2021	28.8	28.9 (27.6-29.8)	-0.3% (-3.4% to 4.5%)	113 (-1689 to 1382)
	Combined	27.6	28.8 (27.8-29.5)	-4.2% (-6.5% to -0.6%)	3308 (439-5247)
Melanoma	2020	18.8	22.2 (21.3-23.0)	-15.4% (-18.4% to -12.0%)	9349 (7037-11613)
	2021	21.7	22.3 (21.1-23.2)	-2.6% (-6.7% to 3.0%)	1613 (-1718 to 4281)
	Combined	20.2	22.2 (21.3-23.0)	-9.0% (-12.0% to -5.0%)	10962 (5875-15088)
Urinary Bladder	2020	17.1	18.4 (18.0-18.9)	-6.8% (-9.2% to -4.9%)	3423 (2423-4769)
	2021	17.6	18.3 (17.8-19.0)	-3.9% (-7.4% to -1.5%)	1984 (730-3885)
	Combined	17.4	18.3 (18.0-18.9)	-5.4% (-8.1% to -3.6%)	5407 (3574-8349)
Non-Hodgkin Lymphoma	2020	17.5	19.0 (18.6-19.3)	-7.5% (-9.3% to -5.7%)	3892 (2889-4926)
	2021	18.2	18.9 (18.5-19.4)	-3.8% (-6.1% to -1.7%)	1975 (876-3271)
	Combined	17.9	18.9 (18.6-19.3)	-5.6% (-7.5% to -4.1%)	5867 (4190-7966)
Kidney and Renal Pelvis	2020	16.2	18.0 (17.6-18.4)	-10.4% (-12.2% to -8.0%)	5151 (3857-6170)
	2021	16.9	18.1 (17.3-18.6)	-6.4% (-9.3% to -2.4%)	3173 (1147-4773)
	Combined	16.5	18.0 (17.5-18.5)	-8.4% (-10.4% to -5.5%)	8324 (5280-10584)
Pancreas	2020	13.2	13.8 (13.4-14.0)	-4.3% (-5.9% to -1.3%)	1628 (481-2257)
	2021	13.5	13.9 (13.3-14.2)	-2.8% (-5.1% to 1.4%)	1058 (-520 to 1978)
	Combined	13.3	13.8 (13.4-14.1)	-3.5% (-5.3% to -0.3%)	2686 (196-4084)

[a] Rates given per 100,000 people in the population and age-adjusted to the 2000 U.S. Standard Population
Abbreviations: PI = Prediction Interval; NOS = Not Otherwise Specified.

© 2024 Burus T et al. *JAMA Network Open.*

eTable 2. Potentially Missed Screening-Detected Cancer Cases by Stage at Diagnosis and Period, 2020-2021, SEER 22 Database, 2001-2021[6]

Site	Period	Observed Rate[a]	Expected Rate (95% PI)	Relative Difference (95% PI)	Potential Missed Cases (95% PI)
Early Stage					
Female Breast	2020	77.1	85.4 (83.0 - 87.9)	-9.7% (-12.3% to -7.1%)	11383 (8048 - 14790)
	2021	88.3	85.8 (83.0 - 88.7)	2.9% (-0.5% to 6.4%)	-3377 (-7328 to 610)
	Combined	82.7	85.6 (83.5 - 87.8)	-3.4% (-5.8% to -1.0%)	8005 (2185 - 13996)
Lung and Bronchus	2020	12.2	14.9 (13.9 - 16.4)	-18.4% (-26.1% to -12.5%)	7550 (4779 - 11764)
	2021	13.2	15.3 (13.9 - 17.2)	-13.7% (-23.5% to -5.1%)	5765 (1927 - 11120)
	Combined	12.7	15.1 (14.0 - 16.6)	-16.1% (-23.6% to -9.5%)	13315 (7266 - 21513)
Colon and Rectum	2020	10.8	12.6 (11.5 - 13.3)	-14.3% (-19.2% to -6.2%)	4926 (1940 - 7019)
	2021	12.4	12.4 (11.1 - 13.3)	0.4% (-6.8% to 11.6%)	-140 (-3535 to 2468)
	Combined	11.6	12.5 (11.5 - 13.2)	-7.0% (-12.3% to 1.0%)	4786 (-601 to 8963)
Cervix Uteri	2020	2.8	3.5 (3.2 - 3.8)	-18.0% (-24.7% to -12.1%)	859 (535 - 1277)
	2021	3.1	3.5 (3.2 - 3.8)	-11.5% (-18.6% to -4.9%)	551 (220 - 968)
	Combined	3.0	3.5 (3.3 - 3.7)	-14.8% (-20.0% to -9.8%)	1410 (886 - 2041)
Late Stage					
Female Breast	2020	40.0	42.2 (40.5 - 43.5)	-5.2% (-8.1% to -1.3%)	2988 (751 - 4810)
	2021	43.1	42.0 (40.4 - 43.4)	2.6% (-0.9% to 6.7%)	-1477 (-3694 to 529)
	Combined	41.5	42.1 (40.8 - 43.4)	-1.3% (-4.2% to 1.9%)	1511 (-2118 to 5046)
Lung and Bronchus	2020	29.5	32.2 (30.3 - 34.4)	-8.5% (-14.4% to -2.9%)	7490 (2420 - 13638)
	2021	29.7	31.7 (29.2 - 34.9)	-6.4% (-15.0% to 1.8%)	5534 (-1474 to 14396)
	Combined	29.6	31.9 (30.0 - 34.5)	-7.4% (-14.3% to -1.4%)	13024 (2278 - 27009)
Colon and Rectum	2020	20.1	21.6 (21.0 - 22.2)	-7.0% (-9.4% to -4.4%)	4132 (2569 - 5709)
	2021	21.9	21.6 (21.0 - 22.2)	1.1% (-1.6% to 4.0%)	-657 (-2288 to 983)
	Combined	21.0	21.6 (21.2 - 22.1)	-2.9% (-4.9% to -0.9%)	3474 (1009 - 5996)
Cervix Uteri	2020	3.8	3.9 (3.7 - 4.1)	-3.5% (-7.9% to 1.2%)	188 (-63 to 439)
	2021	4.0	3.9 (3.7 - 4.1)	3.8% (-1.4% to 9.5%)	-203 (-480 to 80)
	Combined	3.9	3.9 (3.7 - 4.0)	0.1% (-3.7% to 4.3%)	-15 (-435 to 406)

[a] Rates given per 100,000 people in the population and age-adjusted to the 2000 US Standard Population
[b] Early stage at diagnosis defined as localized stage only
[c] Late stage at diagnosis defined as regional and distant stage

Abbreviations: PI = Prediction Interval

© 2024 Burus T et al. *JAMA Network Open.*

eFigure 1. Percentage Difference in Observed From Expected All Sites Cancer Incidence Rates, by Population Subgroup and Period, 2020-2021.[6] Error bars indicate the 95% prediction interval (PI). (*) indicates significantly worse disruption than comparison group based on non-overlapping 95% PIs. NH = Non-Hispanic; API = Asian and Pacific Islander.

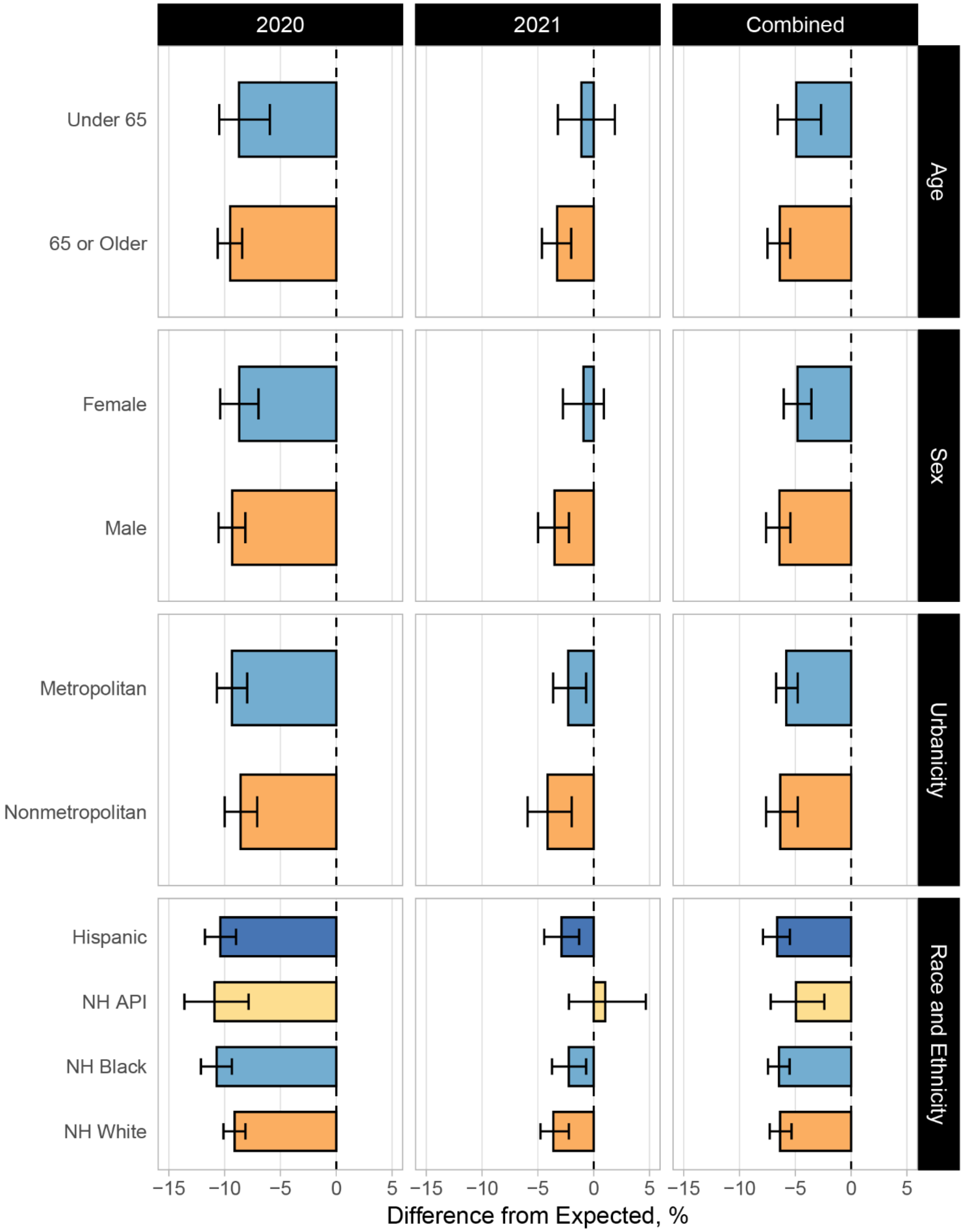

© 2024 Burus T et al. *JAMA Network Open.*

eFigure 2. Percentage Difference in Observed From Expected Incidence Rates, by Urbanicity, Site, and Period, 2020-2021.[6] Error bars indicate the 95% prediction interval (PI). (*) indicates significantly worse disruption than comparison group based on non-overlapping 95% PIs. NOS = Not Otherwise Specified.

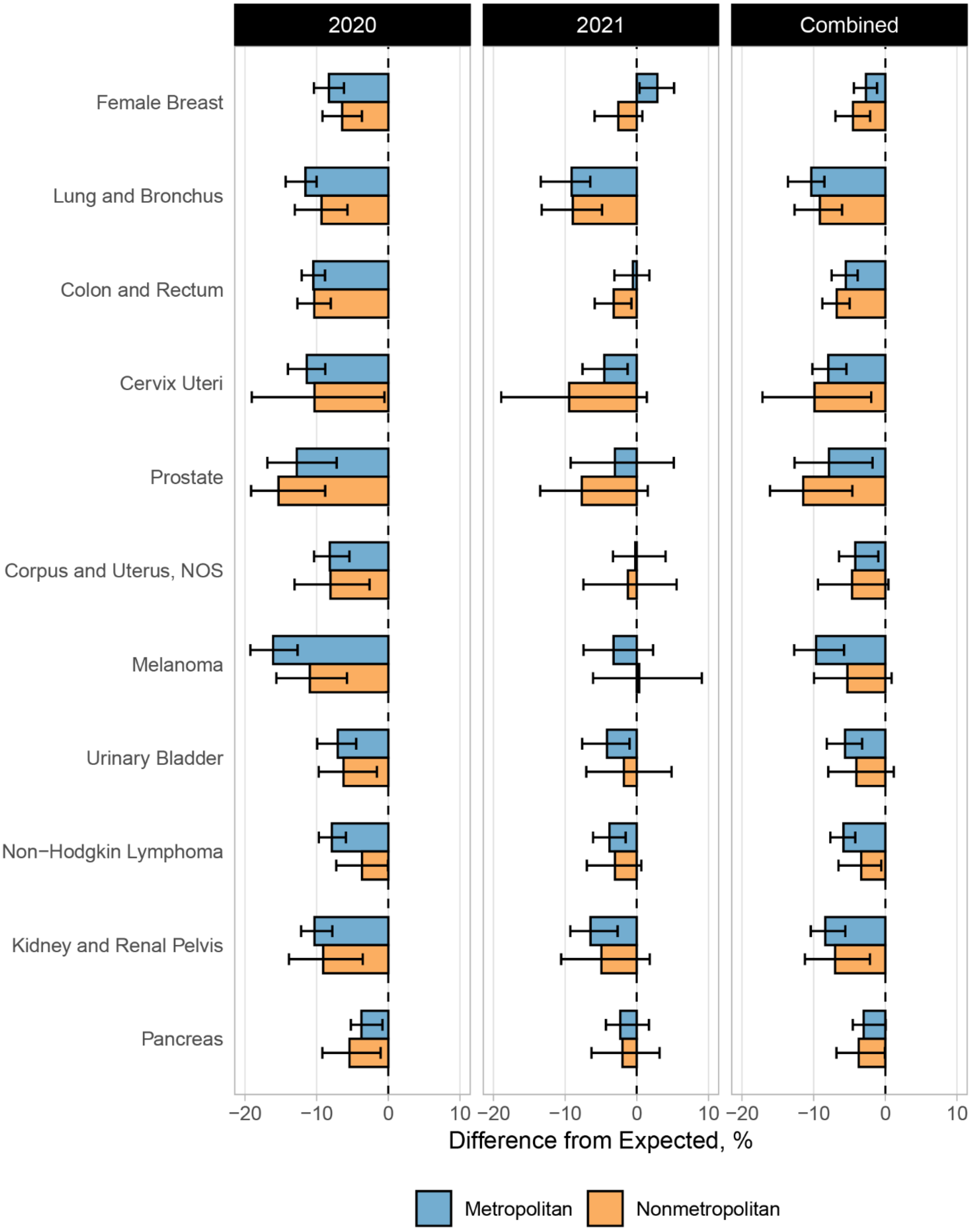

© 2024 Burus T et al. *JAMA Network Open.*

eFigure 3. Percentage Difference in Observed From Expected Incidence Rates, by Age, Site, and Period, 2020-2021.[6] Error bars indicate the 95% prediction interval (PI). (*) indicates significantly worse disruption than comparison group based on non-overlapping 95% PIs. NOS = Not Otherwise Specified**.**

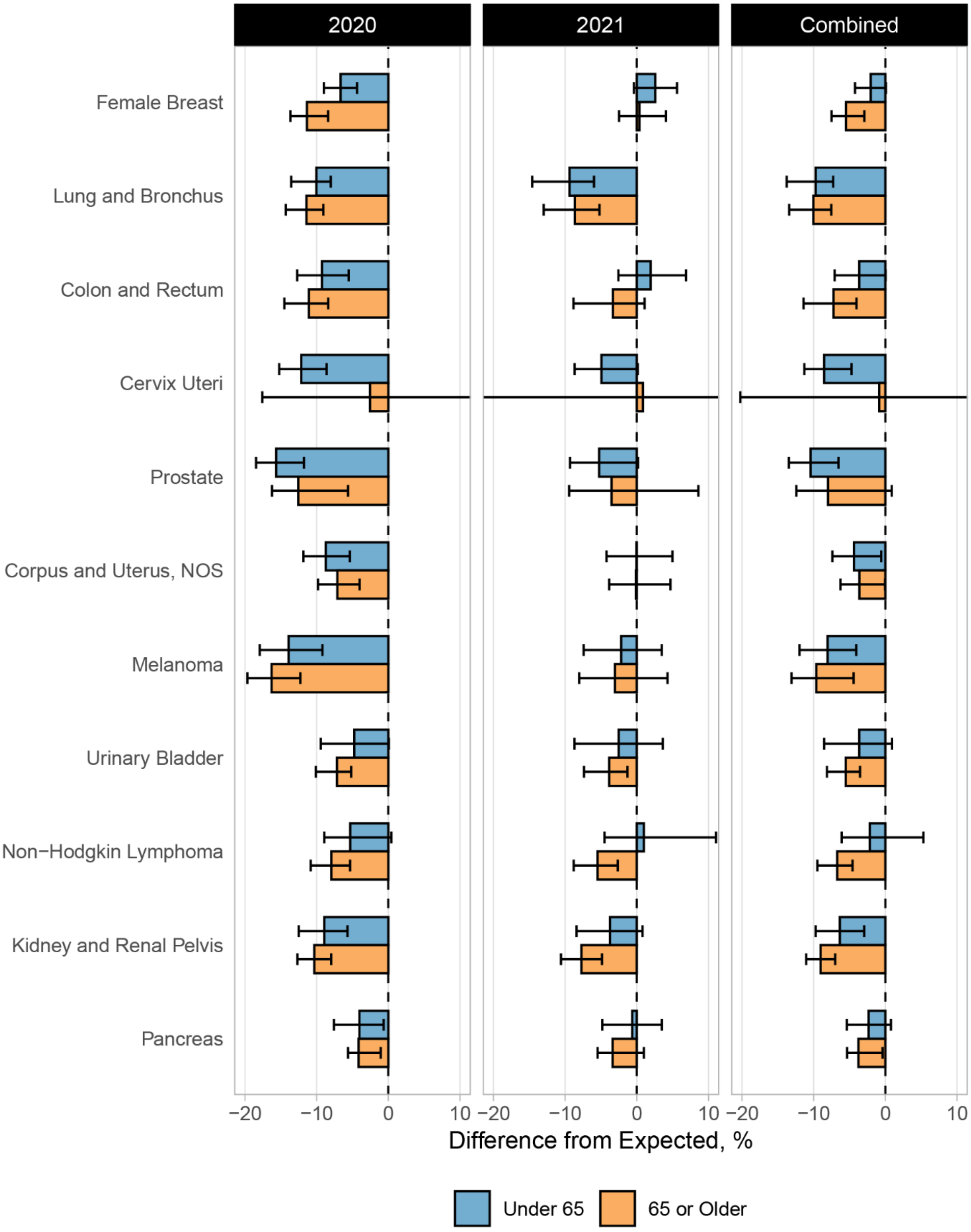

© 2024 Burus T et al. *JAMA Network Open.*

eFigure 4. Percentage Difference in Observed From Expected Incidence Rates, by Race and Ethnicity, Site, and Period, 2020-2021.[6] Error bars indicate the 95% prediction interval (PI). (*) indicates significantly worse disruption than comparison group based on non-overlapping 95% PIs. NOS = Not Otherwise Specified; NH = Non-Hispanic.

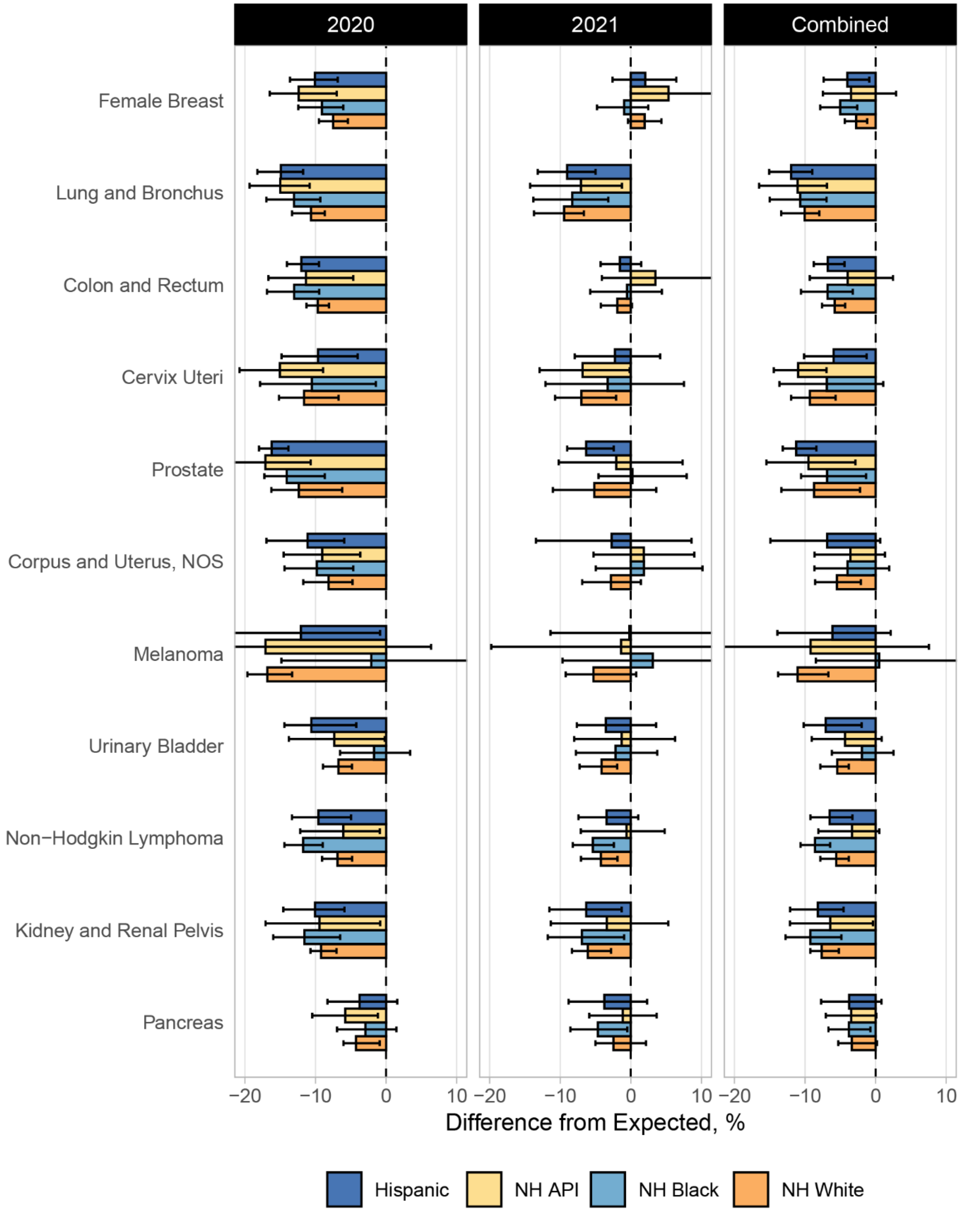

© 2024 Burus T et al. *JAMA Network Open.*

eFigure 5. Percentage Difference in Observed From Expected Incidence Rates, by Sex, Site, and Period, 2020-2021.[6] Error bars indicate the 95% prediction interval (PI). (*) indicates significantly worse disruption than comparison group based on non-overlapping 95% PIs.

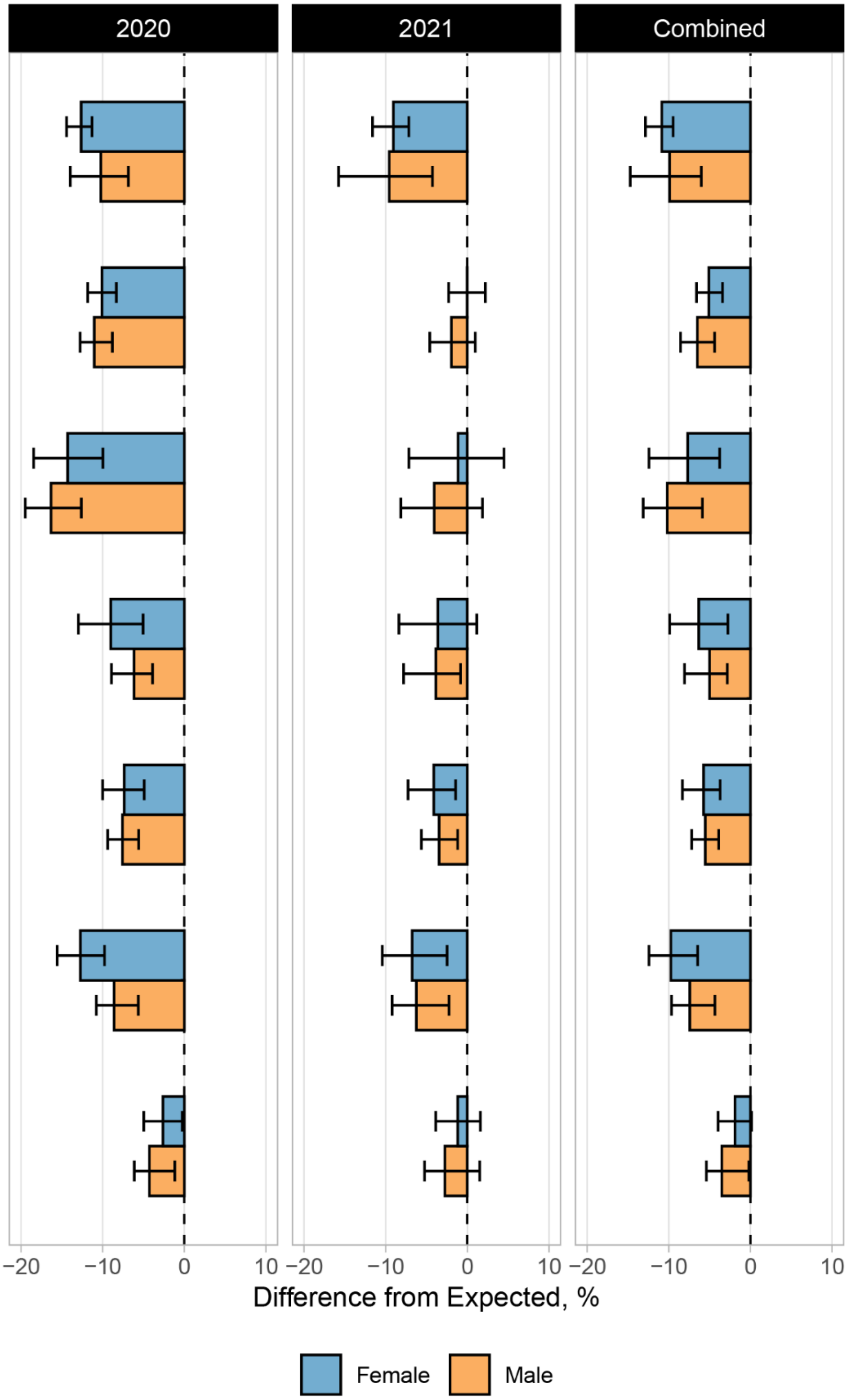

© 2024 Burus T et al. *JAMA Network Open.*

eFigure 6. Percentage Difference in Observed From Expected Incidence Rates, by Sex, Age, Site, and Period, 2020-2021.[6] Error bars indicate the 95% prediction interval (PI).
(*) indicates significantly worse disruption than comparison group based on non-overlapping 95% PIs.

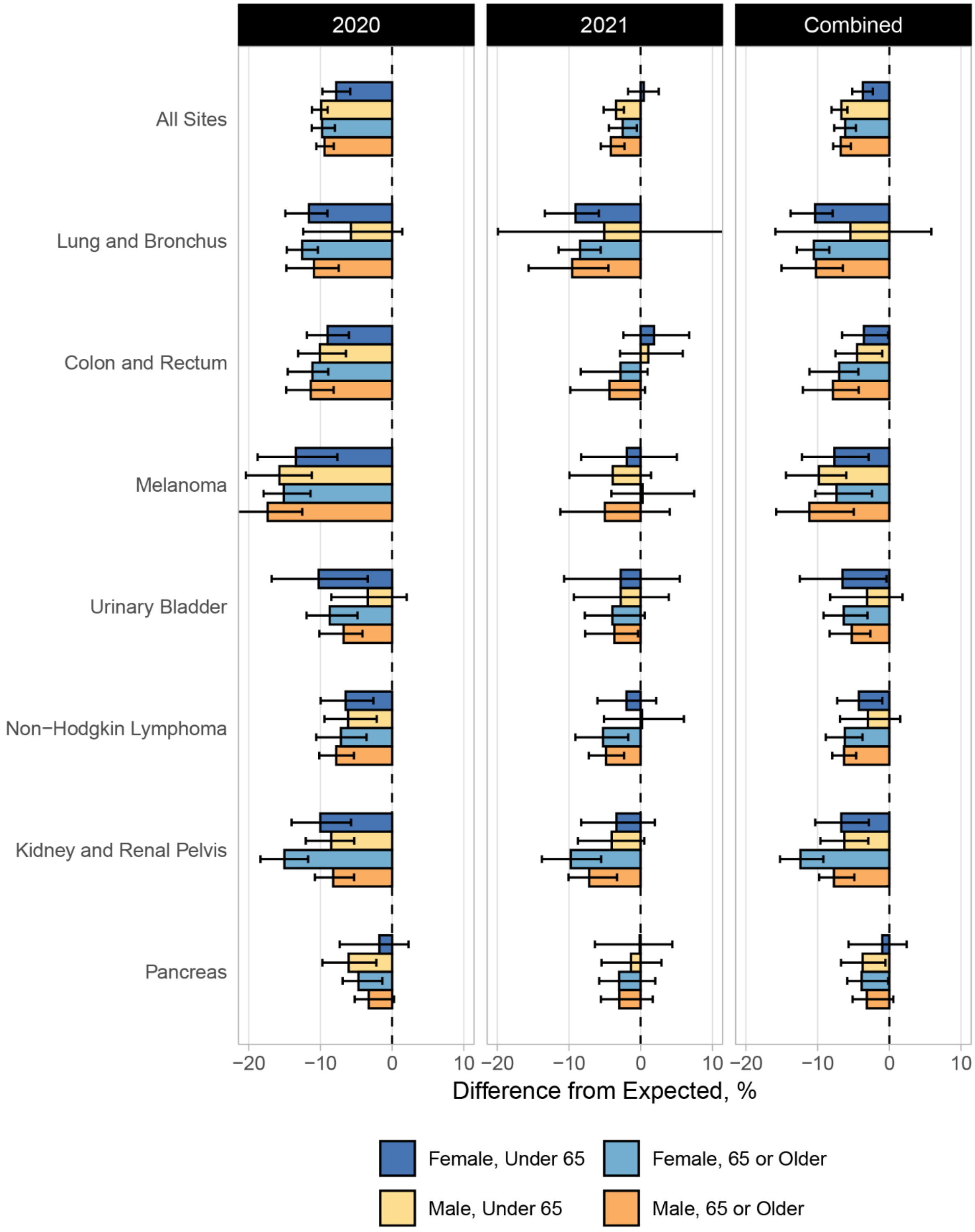

© 2024 Burus T et al. *JAMA Network Open.*

eFigure 7. Monthly Age-Adjusted All Cancer Sites Incidence Rates, by Sex and Age, January 2017-December 2021.[6] (A) Female, under age 65 years; (B) male, under age 65 years; (C) female, age 65 years or older; (D) male, age 65 years or older. Grey segment highlights March-April 2021.

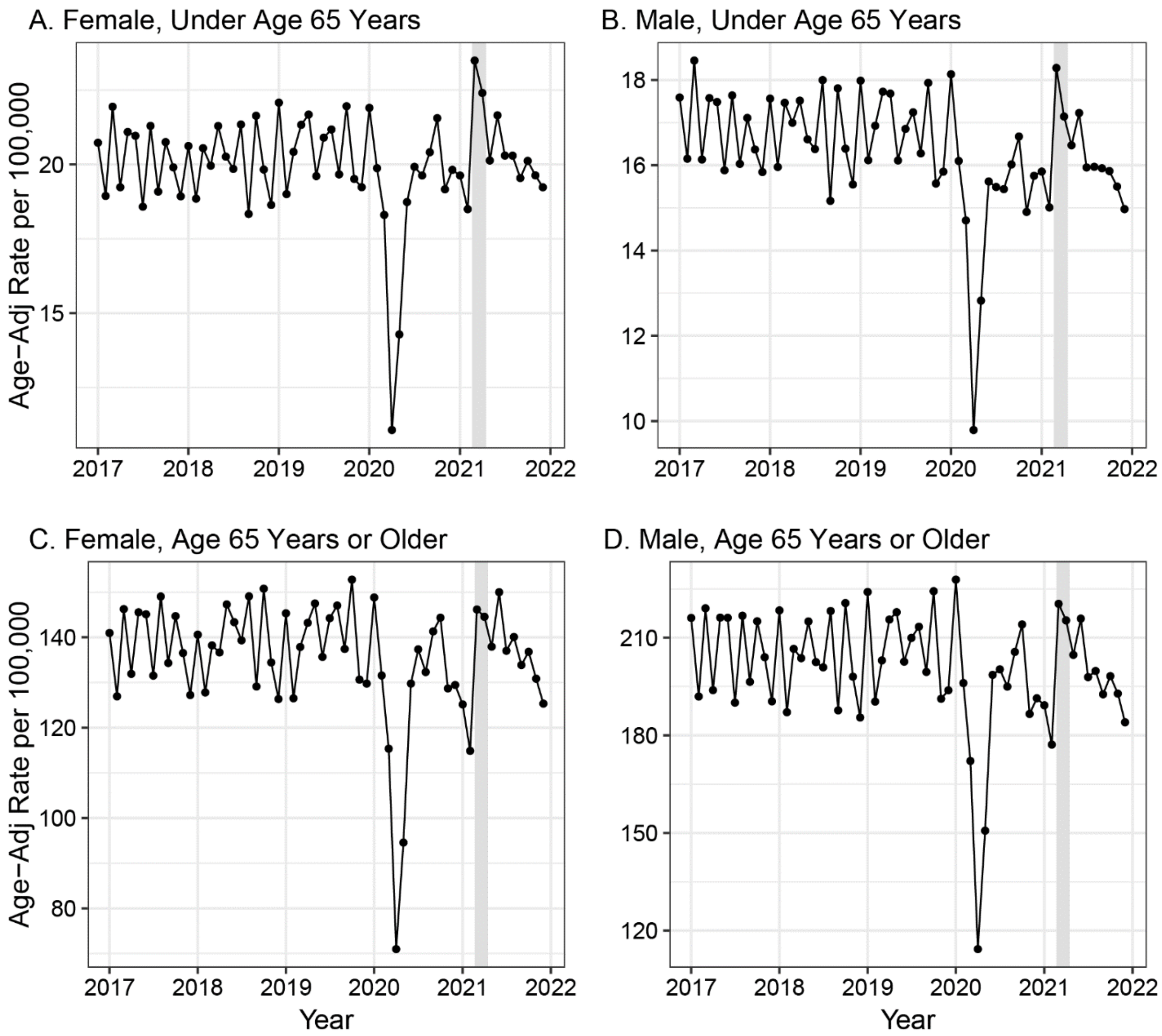

© 2024 Burus T et al. *JAMA Network Open.*

eReferences.

1. Kim HJ, Fay MP, Feuer EJ, Midthune DN. Permutation tests for joinpoint regression with applications to cancer rates. *Statist Med*. 2000;19(3):335-351. doi:10.1002/(SICI)1097-0258(20000215)19:3<335::AID-SIM336>3.0.CO;2-Z

2. Division of Cancer Control & Population Sciences, National Cancer Institute. Method and Parameters Tab. Accessed April 26, 2024. https://surveillance.cancer.gov/help/joinpoint/setting-parameters/method-and-parameters-tab

3. Burus T, Lei F, Huang B, et al. Undiagnosed Cancer Cases in the US During the First 10 Months of the COVID-19 Pandemic. *JAMA Oncol*. Published online February 22, 2024. doi:10.1001/jamaoncol.2023.6969

4. Wallis KF. Combining Density and Interval Forecasts: A Modest Proposal*. *Oxf Bull Econ Stat*. 2005;67(s1):983-994. doi:10.1111/j.1468-0084.2005.00148.x

5. Wang X, Hyndman RJ, Li F, Kang Y. Forecast combinations: an over 50-year review. *International Journal of Forecasting*. 2023;39(4):1518-1547. doi:10.1016/j.ijforecast.2022.11.005

6. National Cancer Institute, DCCPS, Surveillance Research Program. Surveillance, Epidemiology, and End Results (SEER) Program (www.seer.cancer.gov) SEER*Stat Database: Incidence - SEER Research Limited-Field Data, 22 Registries, Nov 2023 Sub (2000-2021). Published online April 2024.

© 2024 Burus T et al. *JAMA Network Open.*
