# Supplementary material for: COVID-19 and Rates of Cancer Diagnosis in the US
Source: JAMA Netw Open. 2024 Sep 6;7(9):e2432288. doi: 10.1001/jamanetworkopen.2024.32288 (PMC11380103; doi:10.1001/jamanetworkopen.2024.32288)
Supplement: Supplement 2. — Data Sharing Statement [file jamanetwopen-e2432288-s002.pdf]

Data Sharing Statement

Burus. COVID-19 and Rates of Cancer Diagnosis in the US. *JAMA Netw Open*. Published September 06, 2024. doi:10.1001/jamanetworkopen.2024.32288

Data
Data available: No

Additional Information
Explanation for why data not available: The data used for this study is from the National Cancer Institute's Surveillance, Epidemiology, and End Results program. Investigators can access this data through a data use agreement with the National Cancer Institute.
